# Salt-Inducible Kinase 2: An Oncogenic Signal Transmitter and Potential Target for Cancer Therapy

**DOI:** 10.3389/fonc.2019.00018

**Published:** 2019-01-22

**Authors:** Fangyu Chen, Liuwei Chen, Qin Qin, Xinchen Sun

**Affiliations:** ^1^Department of Radiation Oncology, The First Affiliated Hospital of Nanjing Medical University, Nanjing, China; ^2^The First School of Clinical Medicine, Nanjing Medical University, Nanjing, China

**Keywords:** salt-inducible kinase, SIK2, cancer, signaling pathway, target therapy

## Abstract

Salt-inducible kinase (SIK), which belongs to the sucrose non-fermenting 1/AMP-activated protein kinase family, was first discovered in the adrenal cortex of a rat on a high-salt diet. As an isoform of the SIK family, SIK2 modulates various biological functions and acts as a signal transmitter in various pathways. Compared with that in adjacent normal tissues, the expression of SIK2 is significantly higher in multiple types of tumors, which indicates its pivotal effect in oncogenesis. Studies on SIK2 have recently underlined its role in several signaling pathways, including the PI3K-Akt-mTOR pathway, the Hippo-YAP pathway, the LKB1-HDAC axis, and the cAMP-PKA axis. Moreover, a few small-molecule SIK2 inhibitors have been found to be able to rescue the oncogenicity of SIK2 during tumor development and reverse its abnormal activation of downstream pathways. In this mini-review, we discuss the results of *in vivo* and *in vitro* studies regarding the SIK2 mechanism in different signaling pathways, particularly their regulation of cancer cells. This work may provide new ideas for targeting SIK2 as a novel therapeutic strategy in tumor therapy.

## Introduction

Plasma ion balances regulate a wide range of cellular processes from cell proliferation to mitochondrial functions. The plasma concentrations of Na+ and K+ have been proven to play a vital role in the biosynthesis of aldosterone in the adrenal cortex. Studies have shown that changes in plasma ion concentration can target biomembrane ion channels, such as Na+-K+-ATPase to regulate extra- and intracellular ion balances (1, 2). As a major part of this ion modulation network, salt-inducible kinase (SIK) was first discovered in 1999 by Okamoto et al. in the adrenal cortex of a rat on a high-salt diet. SIK is a serine/threonine protein kinase that belongs to the sucrose non-fermenting 1/AMP-activated protein kinase (SNF1/AMPK) family. The SIK family comprises three isoforms, namely, SIK1, SIK2, and SIK3, all of which may act as metabolic transmitters. The SIK2 gene is located on chromosome 11 and encodes for the SIK2 protein, which has 926 amino acids and three domains (3, 4). The C-terminal domain of the SIK protein contains numerous unique sites that can be phosphorylated by different protein kinases and transmit various stimulation signals involved in different biological processes, including cell growth and apoptosis (4–8). In many malignant tumors, such as breast cancer, lung cancer, melanoma, primary liver cancer, and ovarian cancer, SIK expression is significantly different from that in adjacent tissues (9–14).

Growing evidence has proven that the expression and action of SIK2 are tissue-specific. The cellular and subcellular distributions of SIK should be considered to determine its mechanism. Earlier investigations demonstrate that SIK2 maintains cell homeostasis via modulation of cAMP response element binding protein (CREB)-mediated gene transcription during starvation, which may be a possible mechanism for cancer cell survival under stress, such as chemoradiotherapy (15). SIK2 reduces glucose uptake in muscle cells and white adipocytes and downregulates lipogenesis and ketogenesis by phosphorylating the glucose-activated histone acetyltransferase coactivator p300 (16). SIK2 modulates several subtle cellular signaling pathways, and its abundant expression in melanoma and ovarian tumors is suggestive of its pivotal function in tumor development (13, 17). Thus, in this mini-review, we discuss the specific role and related signaling pathways of SIK2 in tumorigenesis. Our findings indicate the potential application of SIK2 as a therapeutic target for cancers.

## SIK Family and Their Functions

**Figure 1:**
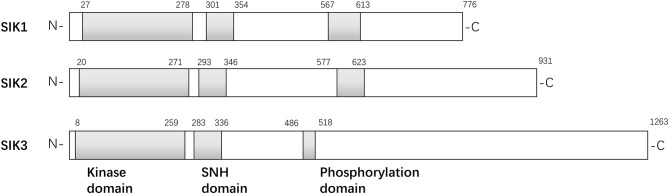
Structures of isoforms in SIK family.

The structures of the SIK isoforms are shown in Figure 1. The three isoforms are similar to one another, particularly in three domains: a kinase domain near the N-terminal, a central SNF1 protein kinase homology (SNH) domain, and a phosphorylation domain near the C-terminal (3). SIK1 is a 776-amino acid protein with a kinase domain in the region of residues 27–278, an SNH domain in the region of residues 301–354, and a domain enriched with PKA-dependent phosphorylation sites in the region of residues 567–613. Similarly, SIK2 is a 931-amino acid protein with a kinase domain in the region of residues 20–271, an SNH domain in the region of residues 293–346, and a phosphorylation domain in the region of residues 577–623. Finally, SIK3 is a 1,263-amino acid protein with a kinase domain in the region of residues 8–259, an SNH domain in the region of residues 283–336, and a phosphorylation domain in the region of residues 486–518. Initial studies have found that SIK1 is most abundant in the adrenal cortex and an important regulator in the early phase of hormonal stimulation of the adrenal cortex (4, 18), adipose tissue (6), and neural tissue (19). It may overexpress in several non-adipose tissues, such as in the ovaries and lungs, and act as an oncogenic signal transmitter during the occurrence and progression of tumors in the aforementioned organs (18–20). Unlike SIK1, SIK2 modulates several subtle cellular signaling pathways, and the increased expression of SIK2 in adipose and neuronal tissues indicates its pivotal role in lipid metabolism and neural physiology. SIK2 promotes insulin resistance and diabetes by reducing glucose uptake in muscles and white adipose tissues and inhibiting gluconeogenesis (7). SIK2 is overexpressed in several cancer cell lines and boosts cancer cell tolerance to different stresses, such as deprivation of nutrients and taxol chemotherapy (21). It plays a proinflammatory role by repressing IL-10 secretion of regulatory macrophages (22). However, little is known about why the structural similarity of the SIK family leads to different biological functions.

## SIK2 and the PI3K-Akt-mTOR Pathway

The expression level of SIK2 in cancers is significantly higher than that in adjacent and surrounding normal tissues, which suggests that SIK2 is critical in tumorigenesis and tumor development. Miranda et al. found that the loss of SIK2 reduces G1/S transition, delays mitotic progression, and decreases Akt phosphorylation levels (17). They also confirmed that SIK2 is overexpressed in adipocyte-rich metastatic deposits compared with ovarian primary lesions and that adipocytes activate SIK2 in ovarian cancer cells in a calcium-dependent manner. Following adipocyte-induced stimulation, the activated SIK2 alters metabolic effects in ovarian cancer cells by inhibiting acetyl-CoA carboxylase and promoting fatty acid oxidation. p85α, the regulatory subunit of the PI3K complex, was previously identified as a putative SIK2 substrate during chemical genetic screening. The identified p85α phosphorylation site (S154) resides in the known SIK2 phosphorylation consensus sequence L-x-[HKR]-[ST]-x-S-X(3)-L at L149–L158 (LYRTQSSSNL). Incubation of recombinant full-length SIK2 or its kinase domain with a peptide corresponding to L149–L158 of p85α confirmed that SIK2 catalyzes the phosphorylation of this sequence. More importantly, full-length SIK2, but not the kinase-inactive mutant, phosphorylated p85α was confirmed in isotopic labeling assay. Phosphopeptide mapping of p85α following incubation with SIK2 (kinase domain or full-length) revealed that the former was phosphorylated at S154 in the BH domain. The BH domain is thought to bind to proteins that modulate PI3K activity. Downstream S154 phosphorylation also appears to increase in an SIK2-dose-dependent manner. siRNA-mediated depletion or chemical inhibition confirms that SIK2 is required for p85α S154 phosphorylation. Moreover, p85α phosphorylation and concomitant Akt phosphorylation can be triggered by calcium-mediated SIK2 activation. Consistent with these observations, incubation of the PI3K complex with recombinant SIK2 leads to a profound increase in PI3K activity *in vitro* (up to 13.8-fold), while chemical inhibition of SIK2 induces a dose-dependent reduction in PI3K activity to its basal level. These data confirm that p85α is a direct catalytic substrate of SIK2 and that SIK2 S154 phosphorylation significantly increases the activity of the PI3K-Akt pathway in ovarian cancer cells.

While most reports suggest that SIK2 is an oncogenic marker, one study in Turkey claimed that SIK2 is a potential tumor suppressor in breast cancer (23); SIK2 expression was reportedly reduced in tumor tissues and breast cancer cell lines compared with that in normal counterparts. The researchers also found SIK2-mediated attenuation of proliferation and survival of breast cancer cells with parallel inhibition of the Ras-Erk and PI3K-Akt pathways. However, the mechanisms underlying the reduction of SIK2 levels in cancer tissues were not discussed. Thus, research into the mechanism of SIK2 loss will help future scholars better understand tumor transformation in breast tissue and design new treatment strategies.

## SIK2 and the Hippo-YAP Pathway

The Hippo pathway is a highly conserved growth regulatory signaling pathway that was first discovered in *Drosophila*. It can block the downstream pro-growth transcriptional co-activator Yorkie (Yki), which is homologous to mammalian Yes-associated protein (YAP), and exert its regulatory effects on organ size, cell proliferation, and apoptosis during organ development (24, 25). YAP has been shown to be highly expressed in various human tumors, such as endometrial carcinoma, primary liver cancer, and oral squamous cell carcinoma. Activation of YAP can remove tumor cell contact inhibition, leading to tumor metastasis (25–27). Tsujiura et al. immunohistochemically analyzed YAP in endometrial carcinoma tissue samples and found that the high expression of YAP in the nucleus is closely associated with higher tumor grading and staging, lymphatic/blood vessel invasion, increased recurrence, and metastasis. They then confirmed these results at the cellular level in knockdown and overexpression assays. Recent studies have demonstrated that YAP restricts the activity of the cell cycle checkpoints ATM and ChK2 to enable cancer cells to enter the cell cycle and mitosis after chemoradiotherapy despite unrepaired DNA damage, resulting in tumor growth, chemoradiotherapy resistance, and ongoing proliferation (28).

**Figure 2:**
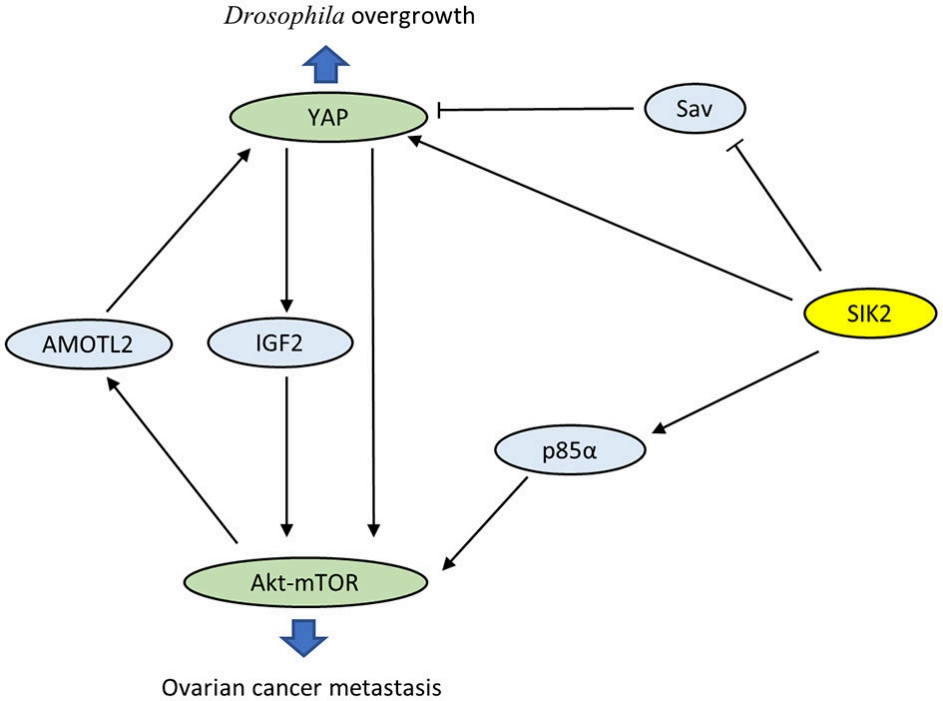
Crosstalk between the PI3K-Akt-mTOR pathway and the Hippo-Yap pathway via SIK2.

Wehr et al. characterized Drosophila salt-inducible kinase (sik2) as an upstream inhibitor of the Hippo pathway (29). sik2 has been identified as the ortholog of human SIK2. Activated sik2 phosphorylates Ser413 of the scaffold protein Salvador (Sav), a major part of the core kinase complex of the Hippo pathway, and subsequently abolishes the inhibition of the proto-oncogene Yki. In addition, sik2 directly induces the expression of Yki and facilitates Yki-dependent tissue overgrowth. Coincidentally, both SIK2 and YAP have been proven to be oncogenes in ovarian cancer. Research has confirmed a close interaction between the PI3K-Akt-mTOR and Hippo-YAP pathways via SIK2 (Figure 2). On the one hand, YAP directly activates PI3K-Akt-mTOR and alters cellular biological functions (30, 31). YAP also increases pAkt-S473 levels and suppresses apoptosis by induction of insulin-like growth factor 2 expression (28). On the other hand, mTOR complex 2 enhances the oncogenicity of YAP through phosphorylation of the Hippo pathway component AMOTL2 (32). These observations reveal that mutual activation between the PI3K-Akt-mTOR and Hippo-YAP pathways caused by SIK2 may be crucial in tumorigenesis. However, the precise role of SIK2 in these intersecting pathways is not well-understood, and future studies are still desperately needed to elucidate the related detailed mechanisms.

## SIK2 and the LKB1-HDAC Signaling Axis

Epigenetic studies have confirmed that DNA acetylation modification is closely related to tumorigenesis, tumor invasion, and chemoradiotherapy resistance (33–35). The abnormal activation and overexpression of histone deacetylase (HDAC) down-regulates tumor suppressor genes and exhibits tumor-promoting effects. Using kinase domain-focused CRISPR techniques, researchers screened all dependent kinase in acute myeloid leukemia (AML), focusing subsequent experiments on SIK3, which scored strongly in MOLM-13 and MV4-11 AML cells and in a more intermediate fashion in other AML cell lines (36). Liver kinase B1 (LKB1) was also identified to show an AML-biased pattern of dependence. Since SIK3 is homologous to SIK1 and SIK2, further studies were conducted to determine whether a broader requirement exists for SIKs in cancer. By performing dual targeting of each SIK gene combination in 17 AML cell lines, researchers observed a broad AML-specific requirement for SIK2 + SIK3 resembling the pattern of LKB1 dependence with a bias for lines with mixed lineage leukemia fusions. In cDNA rescue assays, LKB1 was found to phosphorylate and activate SIK3 in AML. The SIK3 mutant was unable to maintain the proliferation of MOLM-13 cells, while a phosphomimetic allele of SIK3 rescued the proliferation arrest caused by inactivating LKB1. The reverse of SIK3 dependence for AML proliferation was observed during dual CRISPR targeting of HDAC4. Western blotting revealed reductions in HDAC4 phosphorylation upon genetic targeting of SIK3 or chemical inhibition of SIK. Taken together, these results indicate that the function of SIK3 is critical in AML and that inhibition of HDAC4 is one of the key functions of SIK3 in supporting AML proliferation.

Histone H3 lysine 27 acetylation (H3K27ac) is linked to the relevant downstream activity in the LKB1-SIK pathway, and ChIP-seq has confirmed that LKB1/SIK3-dependent H3K27ac coincides with sites of transcription factor MEF2C occupancy. While LKB1/SIK3 knockout or following SIK inhibitor HG-9-91-01 treatment did not change MEF2C protein expression, HG-9-91-01 exposure led to increased HDAC4 binding to MEF2C-bound sites. Epigenomic analysis suggests that LKB1-SIK signaling is critical in AML to prevent HDAC4 from inactivating the function of MEF2C on chromatin. These genetic experiments suggest that co-inhibition of SIK2 + SIK3 could be the ideal strategy to achieve potent MEF2C inhibition in AML. Since MEF2C is maladjusted in lymphoid malignancies, LKB1-SIK signaling is likely to be important in other hematopoietic cancers (37).

## SIK2 and the cAMP-PKA Signaling Axis

**Figure 3:**
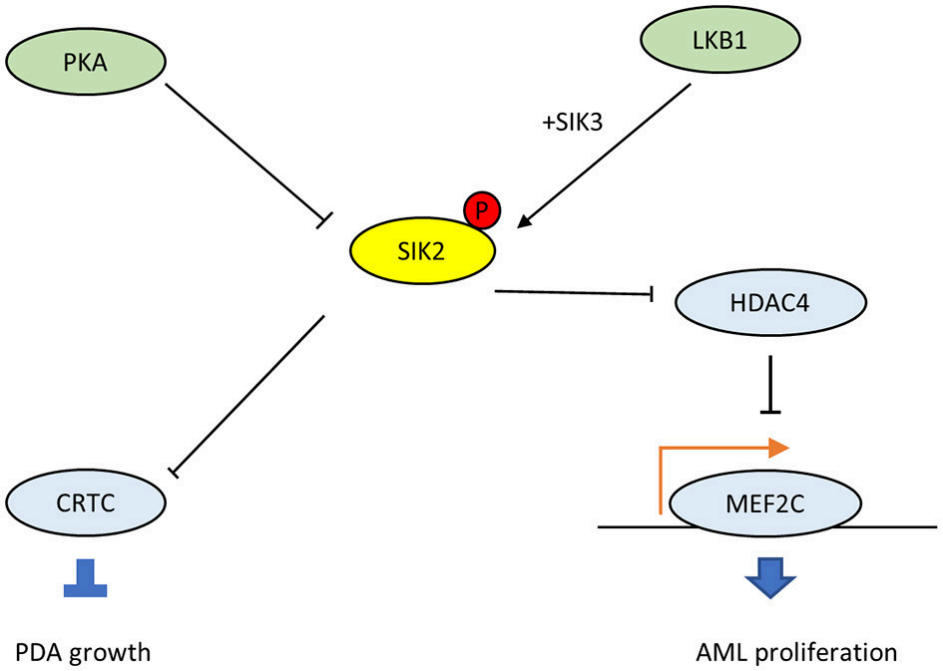
The dichotomous oncogenic roles of SIK2 in the LKB1-HDAC axis and the cAMP-PKA axis.

The G protein αs (GNAS) gene encodes the Gαs stimulatory subunit of G proteins, which mediate G-protein-coupled receptor signaling, a major mechanism that links multiple environmental stimuli with intracellular responses (38). The primary target is adenylyl cyclase, which generates the second messenger cAMP, which, in turn, activates downstream protein kinase A (PKA). In many tissues, GNAS–cAMP-PKA signaling is required during cell dormancy and cell growth (39–43). However, multiple types of human cancers show gain-of-function variations in this pathway (38). For example, loss of p53 promotes the advent of GNAS R201C mutations and induces malignant transformation in pancreatic benign tumors in the KGC mice model, which can rapidly develop cystic pancreatic tumors (44–47). Mutated GNAS R201C supports pancreatic tumor growth via cAMP-PKA signaling, which subsequently phosphorylates SIKs (SIK1, SIK2, and SIK3) and prevents them from phosphorylating downstream targets (48). Also, small molecule pan-SIK inhibitors (HG-9-91-01 and KIN-112) prevent the growth of KGC organoids after silencing GNAS, and their effects are directly proportional to the degree of SIK inhibition. Compared with wild-type SIK2, the SIK2-S4A mutant, which is resistant to cAMP-PKA activation, strongly inhibits the proliferation of KGC-like organs. In particular, SIK^KO^ rescues both organoid growth *in vitro* and subcutaneous tumor growth following GNAS R201C silencing, and these findings have been confirmed in human pancreatic ductal adenocarcinomas (PDA). Thus, the cAMP-PKA-SIK2 signaling pathway is a conserved tumorigenic mechanism in pancreatic tumor cells. The mutant GNAS drives downstream PKA-SIK2 axis and promotes lipid hydrolysis in addition to lipid synthesis and remodeling. While SIK2 is known to maintain cell homeostasis and energetic metabolism, particularly glucose and fatty acid oxidation (15), the suppression of SIK2 mediated by GNAS-PKA will inhibit the phosphorylation of its downstream CREB-regulated transcription co-activator (CRTC) and others (Figure 3). Then it will promote lipids absorption and synthesis, and the abundant lipids in tumor cells provide substrates for structural, signaling, and metabolic purposes, which explains why SIK2 act as a tumor suppressor in PDA.

While SIK2 is deemed to be a tumor promoter in most cases, in the context of GNAS mutated PDA, it is supposed to be a tumor suppressor, mainly because SIK2 plays different roles in different tissue and cells, similar to cAMP/PKA signaling. Given the context-dependent tumor-promoting and -suppressing roles of SIK2, administration of SIK2 inhibitors in GPCR-mutated or other overactive cAMP-PKA cancer types should be attempted with extremely caution to avoid potential pro-tumor effects. More investigations are necessary to clarify these issues and promote the use of SIK2 inhibitors in tumor therapy.

## SIK2 in Cancer Therapy

Previous studies on SIK2 have reported its regulation of energetic metabolism, mostly based on its signaling pathways and the downstream role of LKB1 in adipocytes. Studies on SIK2 have recently underlined its role in several signaling pathways related to tumorigenesis. Clinical and pathological data indicate that SIK2 is a potential oncogenic marker in ovarian (17, 49), prostate (50), osteosarcoma (51), and colorectal (52) cancers by controlling different cellular mechanisms. Intriguingly, two studies report that SIK2 may act as a tumor suppressor in breast cancer and PDA. Since SIK2 plays a distinct role in different tissues and divergent pathways, its dysregulation may lead to conflicting phenotypes. Initial studies on SIK2 maily focused on its role in energetic metabolism, particularly in glucose, and lipids oxidation during starvation. The functions of SIK2 may be unique in cells that are involved in glycolipid metabolism, such as hepatocyte and pancreatic cells. As a consequence, SIK2 may act as both tumor promoter and suppressor due to the diversity of cancer cell types or different genetic background. The SIK2 inhibitors HG-9-91-01, ARN-3236, and KIN-112 have succeeded in cancer therapy approaches, validated in cultured cells and *in vivo* animal models (17, 36, 48), although additional optimization of these small molecules is required for therapeutic investigation. Further evaluation of these small molecules is necessary to achieve potent SIK2 inhibition in the uncontrolled signaling pathways of tumor cells while preserving the homeostatic and tumor-protective functions of SIK2 in other cell types.

## Conclusion

In this mini-review, we discussed the role of the newly identified protein kinase, SIK2, in tumorigenesis, specifically focusing on different signaling pathways involving SIK2. SIKs present significant physiological functions, including novel roles in tumorigenesis and tumor progression. While most studies reveal SIK2 to be a tumor promoter, some claims indicate that SIK2 provides protection from cancer. Thus, the dichotomous function and mechanism between SIK2 and cancer must be further elucidated. As described earlier, SIK2 targeting may be applied as a novel strategy for treating multiple cancer types. Future studies to investigate the molecular mechanisms underlying the precise role of SIK2 in intersecting signaling pathways, as well as the therapeutic effects of SIK2 in preclinical and clinical trials, are recommended.

## Author Contributions

FC, LC, and QQ contributed to conception and manuscript writing. XS participated in its coordination and modification. All authors read and approved the final manuscript.

### Conflict of Interest Statement

The authors declare that the research was conducted in the absence of any commercial or financial relationships that could be construed as a potential conflict of interest.
